# Sequential Obtention of Blood–Brain Barrier-Permeable Non-Polar and Polar Compounds from *Salvia officinalis* L. and *Eucalyptus globulus* Labill. with Neuroprotective Purposes

**DOI:** 10.3390/ijms26020601

**Published:** 2025-01-12

**Authors:** Enrico Romano, Gloria Domínguez-Rodríguez, Luisa Mannina, Alejandro Cifuentes, Elena Ibáñez

**Affiliations:** 1 Food Chemistry Lab, Department of Chemistry and Technology of Drugs, Sapienza University of Rome, P. le Aldo Moro 5, 00185 Rome, Italy; e.romano@uniroma1.it (E.R.); luisa.mannina@uniroma1.it (L.M.); 2 Laboratory of Foodomics, Institute of Food Science Research, CIAL, CSIC, Nicolás Cabrera 9, 28049 Madrid, Spain; elena.ibanez@csic.es; 3 Departamento de Química Analítica, Química Física e Ingeniería Química, Facultad de Ciencias, Universidad de Alcalá, Ctra. Madrid-Barcelona Km. 33.600, Alcalá de Henares, 28871 Madrid, Spain

**Keywords:** *Eucalyptus globulus*, *Salvia officinalis*, SC-CO_2_ extraction, PLE-NaDES, terpenoids, phenolic compounds, neuroprotection, blood–brain barrier

## Abstract

This study investigates the biorefinery approach to extracting blood–brain barrier (BBB)-permeable compounds from *Eucalyptus globulus* Labill. and *Salvia officinalis* L. for neuroprotective purposes. A sequential extraction process was applied, starting with supercritical CO_2_ extraction (SC-CO_2_) to obtain non-polar terpenoids, followed by pressurized natural deep eutectic solvent extraction (PLE-NaDES) to recover phenolic compounds from the SC-CO_2_ residue. PLE-NaDES extracts exhibited higher antioxidant and anticholinergic capacities than SC-CO_2_ extracts for both plants, with *S. officinalis* extracts being more bioactive than *E. globulus* extracts. A total of 21 terpenoids were identified using gas chromatography–mass spectrometry from *E. globulus* while 24 were detected from *S. officinalis* SC-CO_2_ extracts. In addition, 25 different phenolic compounds were identified in both plants using high-performance liquid chromatography coupled with mass spectrometry from PLE-NaDES extracts. The study of the permeability across the BBB showed limited permeability for non-polar compounds obtained by SC-CO_2_ from both plants; however, the more polar compounds obtained by PLE-NaDES showed high permeability, particularly for flavonoids in *E. globulus* and rosmarinic acid in *S. officinalis*. This study revealed, for the first time, the antioxidant and neuroprotective potential of *S. officinalis* and *E. globulus* extracts obtained using SC-CO_2_ followed by PLE-NaDES, as well as the high permeability of PLE-NaDES extracts when crossing the BBB to exert their protective effects. This research opens a new pathway for exploring alternatives to current drugs used in treating neurodegenerative diseases.

## 1. Introduction

Medicinal plants are widely used in phytotherapy because they present many biological activities with health benefits [1]. The biological effects of medicinal plants are attributed to essential oils, phenolic compounds, minerals, and vitamins, among others [2]. Essential oils are a mixture of aromatic and volatile compounds from plants that have been included in the cosmetic and food industries as preservatives and natural flavorings due to their biological capacities. In fact, essential oils exert interesting antimicrobial, antioxidant, anti-inflammatory, anxiolytic, and sedative properties [3]. In particular, terpenoids, which are the major component of essential oils, have an important role in improving resistance against abiotic and biotic stresses in plants. In addition, terpenoids from plants have demonstrated neuroprotective effects in various in vitro and in vivo investigations [4]. The neuroprotective effects exhibited by terpenoids are related to their acetylcholinesterase (AChE) inhibition capacity for the prevention of Alzheimer’s disease (AD) [5]. AD brains have highly altered cholinergic systems with depleted acetylcholine (Ach) levels. Thus, one of the most used treatments for AD is the use of AChE inhibitors to prevent ACh depletion. In addition, butyrylcholinesterase (BChE) is also involved in the hydrolysis of AChE during the last stages of AD, where the levels of AChE are reduced. In this sense, the dual inhibition of AChE and BChE is usually studied, as well as the important ability that these neuroprotective compounds possess to cross the blood–brain barrier (BBB), enabling them to reach the central nervous system and perform their functions [6,7]. Currently, there are synthetic drugs approved by the US Federal Drug Administration (FDA) for AD treatment (such as galantamine and donepezil); however, these agents have multiple side effects. Therefore, to mitigate these negative effects of synthetic drugs, the World Health Organization has recommended the development of safe natural drugs from medicinal plants [6].

Over time, conventional extraction techniques have been employed to recover non-polar compounds, such as terpenoids, from plant matrices for pharmaceutical, food, and cosmetic purposes. The most used are maceration, decoction, Soxhlet extraction, and hydrodistillation [8]. Nevertheless, these extraction techniques require long extraction times and a large amount of solvents to extract target compounds, making them environmentally unsustainable.

Thus, advanced extraction techniques are becoming more popular due to their efficiency in maximizing yield, reducing processing time, enhancing product quality, and offering eco-friendly benefits. In particular, supercritical fluid extraction, where CO_2_ is employed as the extraction solvent (SC-CO_2_), is one of the most environmentally friendly techniques used for extracting volatile compounds such as bioactive terpenoids. It offers high efficiency and selectivity, as the tunable pressure and temperature allow for the precise extraction of desired compounds while minimizing impurities. Unlike conventional extraction techniques, SC-CO₂ provides an inert environment and leaves no toxic residues, ensuring safer extracts for food or pharmaceutical products. Its operation at low temperatures prevents the thermal degradation of heat-sensitive compounds and oxidative damage, preserving their bioactivity and quality, which are advantages that conventional techniques do not offer. Additionally, SC-CO₂ is environmentally friendly, utilizing recyclable CO₂ instead of harmful organic solvents. The process is also faster, scalable, and ideal for extracting volatile compounds without loss or alteration, making it a versatile and sustainable extraction technique [9,10,11]. In particular, *E. globulus* and *S. oficinalis* plants have been shown to be an interesting source of terpenoids, which have been obtained by SC-CO_2_ using temperatures from 40 to 60 °C and extraction pressures ranging from 100 to 220 bar [12,13,14,15]. However, more polar compounds, such as phenolic compounds, could be retained in the residue of SC-CO_2_, requiring solvents that are more polar than CO_2_ for their recovery. The obtention of these compounds would be valuable to fully exhaust the raw material, implementing a biorefinery process. In this sense, Kraujalis et al. [16] and Bendif et al. [17] developed a sequential extraction process using SC-CO_2_ for the recovery of non-polar compounds such as tocopherols, followed by the extraction of phenolic compounds from the SC-CO_2_ residue from *Viburnum opulus* L. and *Thymus munbyanus*, using pressurized liquid extraction (PLE) and employing ethanol as solvent. The interest in extracting phenolic compounds from medicinal plants arises from their strong antioxidant potential for oxidative stress prevention. In fact, phenolic compounds from medicinal plants such as *S. officinalis* and *E. globulus* have shown great antioxidant potential [18,19]. Oxidative stress, along with cholinergic dysfunction, is a major factor that contributes to AD [6,20]. However, despite the antioxidant potential of phenolic compounds from *S. officinalis* and *E. globulus*, their neuroprotective potential has been scarcely studied.

Ultrasound-assisted extraction (UAE), microwave-assisted extraction (MAE), or PLE have been widely employed with water, ethanol, methanol, or a mixture of these, for the extraction of phenolic compounds from several natural matrices [21]. Nevertheless, in the last few years, natural deep eutectic solvents (NaDES) have emerged as a new class of green solvents that provide higher extraction efficiencies than traditional solvents such as ethanol/water. NaDES are based on a mixture of two or more organic components whose melting points are significantly lower than each individual component [22]. In particular, NaDES composed of choline chloride, sugars, polyols, and organic acids are frequently used because these components form strong hydrogen bonds with the hydroxyl groups of phenolic compounds, improving their release into the extraction solvent [23,24]. NaDES have been used for the recovery of phenolic compounds from several matrices by maceration, as well as in combination with advanced extraction techniques such as UAE. To a lesser extent, they have also been included in PLE for the extraction of phenolic compounds from pomegranate peels, avocado residues, soy by-products, and tangerine leaves [23,25,26,27]. It has been observed that the use of NaDES combined with advanced extraction techniques increases the extraction yields of bioactive phenolic compounds compared to the use of NaDES in maceration or the application of advanced extraction techniques with conventional solvents. In addition, NaDES exhibit a protective effect against the degradation of phenolic compounds compared to the use of conventional solvents such as ethanol, water, or their combinations. This protective effect is attributed to the unique physicochemical properties of NaDES, including their high viscosity, hydrogen bonding networks, and the ability to create a stabilizing microenvironment. These characteristics help to shield phenolic compounds from oxidation, hydrolysis, and thermal degradation during extraction, preserving their bioactivity and stability more effectively than conventional solvents [23,24,25,26,27]. Nevertheless, to our knowledge, there are no studies on the recovery of phenolic compounds from medicinal plants, such as *S. officinalis* and *E. globulus*, using PLE combined with NaDES (PLE-NaDES).

Considering the bioactive potential of volatile and non-volatile compounds from medicinal plants, it is essential to implement efficient and sustainable extraction techniques to explore, in detail, all their compounds and fully exploit their bioactive properties. In particular, the research on the medicinal properties of many plants with regard to AD remains limited. Therefore, the aim of this work was to study the potential of terpenoids and phenolic compounds from *Eucalyptus globulus* Labill. and *Salvia officinalis* as antioxidant and neuroprotective agents. To achieve this, a sequential extraction of terpenoids using SC-CO_2_ was carried out, followed by the extraction of phenolic compounds from their SC-CO_2_ residues by PLE-NaDES for the evaluation of their antioxidant capacity, as well as their inhibitory capacity against AChE and BChE enzymes involved in AD. In addition, terpenoid fractions were concisely characterized by gas chromatography with electron impact (EI) single quadrupole (Q) mass spectrometry (GC-EI-Q-MS), while phenolic fractions were characterized by high-performance liquid chromatography (HPLC) with a photodiode array detector (DAD) and electrospray ionization (ESI) quadrupole-time-of-flight (QTOF) mass spectrometry (HPLC-DAD-QTOF-MS). Additionally, to assess the permeability of terpenoid and phenolic compounds across the BBB, the parallel artificial membrane permeability assay for the BBB (PAMPA-BBB) was carried out.

## 2. Results

### 2.1. Bioactive Determination, Characterization, and BBB Permeability of Non-Polar Fractions Obtained from E. globulus and S. officinalis Leaves

The bioactivity of non-polar compounds obtained by SC-CO_2_ from *E. globulus* and *S. officinalis* was evaluated in terms of antioxidant capacity and neuroprotection.

**Figure 1:**
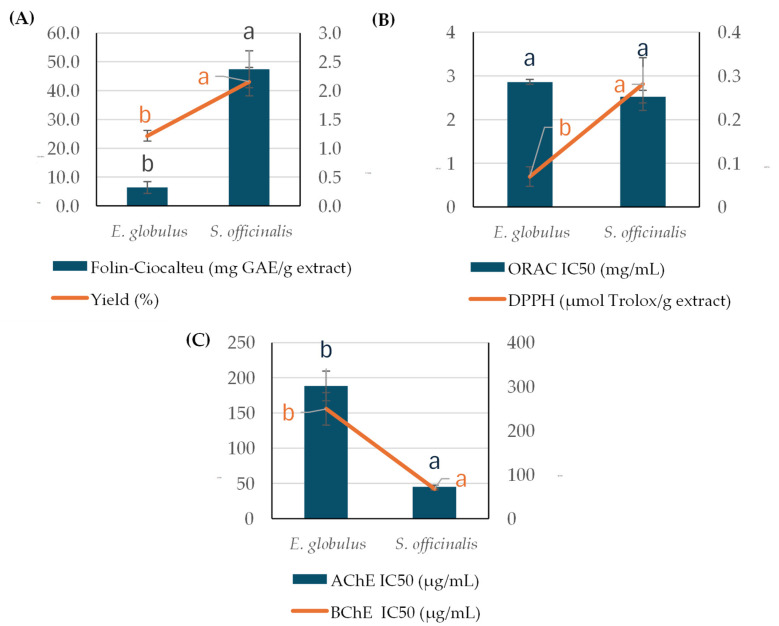
(**A**) TPC, % yield, (**B**) antioxidant capacity determined by ORAC and DPPH assays, and (**C**) anticholinergic capacity evaluated by AChE and BChE assays of SC-CO_2_ extracts obtained from *E. globulus* and *S. officinalis*. a, b. Letters show the significant differences between extracts (*p* ≤ 0.05).

As can be observed in Figure 1, when comparing the extraction yields, SC-CO_2_ *S. officinalis* extracts showed higher values (almost double) than SC-CO_2_ *E. globulus* extracts. It was noted that higher extraction yields provided extracts with higher TPC values (Figure 1A). In addition, *S. officinalis* SC-CO_2_ extracts showed higher antioxidant and anticholinergic capacities than *E. globulus* SC-CO_2_ extracts (Figure 1B,C). In fact, *S. officinalis* extracts showed up to four times more anticholinergic capacity than *E. globulus*.

Concerning non-polar compounds, SC-CO_2_ extracts were characterized by GC-MS. A total of 21 compounds were tentatively identified in *E. globulus* (Table 1). Terpenoids were the majority class of compounds found in *E. globulus* SC-CO_2_ extracts. Seven monoterpenes, three diterpenes, and nine sesquiterpenes were found in this plant. Among all terpenoid classes, the highest diversity was observed within the sesquiterpene class. However, the phytol diterpene (number 19, Figure S1A) was discovered to be the compound with the majority peak area detected in *E. globulus* SC-CO_2_ extracts. This compound was identified with an *m*/*z* ion at 296 and with fragment ions at 278 [M-H_2_O], resulting in an alkene ion that is fragmented, providing ions fragment ions at 123 and 71 [28]. After phytol, spathulenol (number 9, Figure S1B) was also found with the highest peak area in *E. globulus*. This sesquiterpene showed an *m*/*z* ion at 220, with fragment ions at 205 [M-CH_3_] and subsequent characteristic fragment ions at 159, 119, and 91 [29].

Additionally, two non-terpenoid compounds were identified in *E. globulus* SC-CO_2_ extracts that corresponded to α-tocopherol (number 20, Figure S1C) and β-sitosterol (number 21). α-Tocopherol was identified with an *m*/*z* ion at 430 and its characteristic fragment ion at 165, which was generated by the breaking of the chromanol ring structure and the loss of the CH_3_C≡CH fragment. In addition, α-tocopherol exhibited a fragment ion at 205 that corresponded to the loss of the saturated hydrophobic side chain [30]. Furthermore, β-sitosterol was detected with a molecular ion at *m*/*z* 414, with 396 [M-H_2_O], 329 [M-CH_3_-CH-CH_2_-C-CH], 303 [M-CH-CH], and 255 [M-CH_3_-CH_3_-H_2_O] as fragment ions [31].

On the other hand, it was observed that of the twenty-one compounds identified in *E. globulus* SC-CO_2_ extracts, only six crossed the BBB, corresponding to the sesquiterpene family, apart from acetoxy-kauranal diterpene, which also crossed the BBB (Table 1). Among compounds that were able to permeate the BBB, caryophyllene oxide stood out, with 30% of the content obtained in the SC-CO_2_ extracts able to cross the barrier. However, no more than 18% of the rest of the permeable compounds were able to cross the barrier. 

Regarding *S. officinalis* SC-CO_2_ extracts, the two non-terpenoid compounds (α-tocopherol and β-sitosterol) identified in *E. globulus* extracts were also observed in *S. officinalis* SC-CO_2_ extracts (Table 2). In fact, five other compounds identified in *E. globulus* were detected in *S. officinalis*, namely camphene, spathulenol, patchulane, viridiflorol, and phytol. In general, *E. globulus* SC-CO_2_ extracts presented higher peak areas of these compounds than *S. officinalis* SC-CO_2_ extracts, except for camphene and viridiflorol, which were detected with higher peak areas in *S. officinalis* extracts.

Table 2 shows that six monoterpenes, six diterpenes, and nine sesquiterpenes were detected in *S. officinalis* SC-CO_2_ extracts. Similarly to *E. globulus*, the terpenoid class, with highly diverse identified compounds, in *S. officinalis* corresponded to the sesquiterpenoid class. Nevertheless, the most abundant terpenoids (with higher peak areas) detected in *S. officinalis* SC-CO_2_ extracts were diterpenes. In particular, a molecular ion at *m*/*z* 290 was observed, with principal fragment ions at 272 [M-H-H_2_O] and 257 [M-CH_3_-H_2_O] and other minor fragment ions such as 189 [M-H_2_O-C_5_H_11_], 177 [M-H_2_O-C_7_H_11_], and 161 [M-H_2_O-C_7_H_13_]. This was identified as manool (number 16, Figure S2A), with the highest peak area of terpenoids detected in *S. officinalis* SC-CO_2_ extracts. After manool, hinokione (number 20, Figure S2B) was detected as one of the majority compounds with a molecular ion at m/z 300, which provided fragment ions at 272 [M-H_2_O], 257 [M-H_2_O-CH_3_], and 204 [M-C_5_H_8_], as well as smaller hydrocarbon fragments at 121 and 69. In addition, phytol (number 17, Figure S2C) was also observed in S.officinalis as one of the compounds with the highest peak areas, although this was lower than in *E. globulus* SC-CO_2_ extracts. Of the twenty-five compounds identified in *S. officinalis* by GC-MS, only three crossed the BBB: camphene (number 5), totarol (number 18), and hinokione (number 20). Camphene was the most BBB-permeable compound, with 50% of the content from the SC-CO_2_ extract able to cross, while for the rest of the permeable compounds, no more than 25% of the content was able to cross the BBB. 

### 2.2. Bioactivity, Characterization, and BBB Permeability of Phenolic Compounds Obtained by Pressurized NaDES Extraction from the Residue of SC-CO_2_ Extraction

From the residue of SC-CO_2_ extraction, PLE extraction combined with NaDES was carried out to obtain bioactive phenolic compounds.

**Figure 2:**
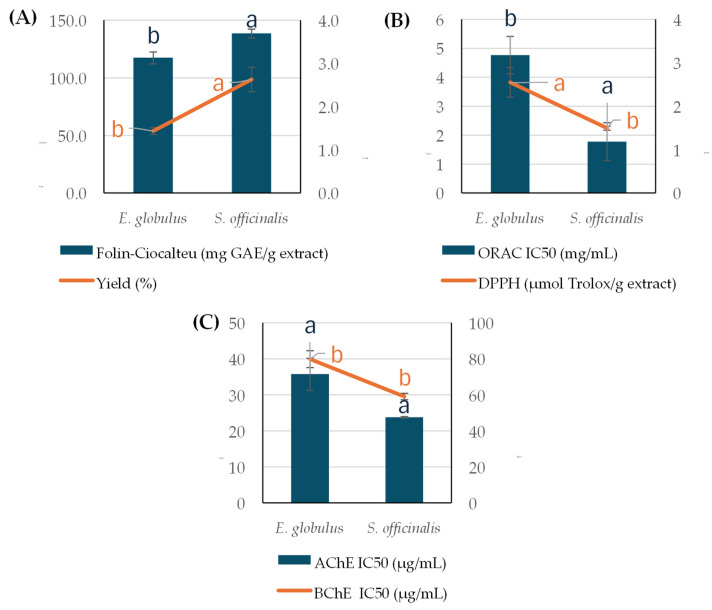
(**A**) TPC, % yield, (**B**) antioxidant capacity determined by ORAC and DPPH assays, and (**C**) anticholinergic capacity evaluated by AChE and BChE assays of PLE-NaDES extracts obtained from *E. globulus* and *S. officinalis*. a, b. Letters show the significant differences between extracts (*p* ≤ 0.05).

As can be observed in Figure 2, PLE-NaDES extracts obtained from *S. officinalis* presented higher TPC values and higher antioxidant and anticholinergic capacities than *E. globulus*. In addition, it was observed that higher TPC values and bioactivity of the extracts implied higher extraction yields.

When comparing SC-CO_2_ and PLE-NaDES extracts (Figure 1 and Figure 2), it is noted that PLE-NaDES provided the extracts with the highest TPC values and the highest anticholinergic capacity. However, the antioxidant capacity depended on the type of assay employed for its determination, as well as the type of medicinal plant. In fact, a higher antioxidant capacity was observed in PLE-NaDES extracts when DPPH was used for both plants. Nevertheless, SC-CO_2_ extracts from *E. globulus* presented a higher antioxidant capacity than PLE-NaDES extracts evaluated using the ORAC assay, while PLE-NaDES extracts obtained from *S. officinalis* showed a higher capacity to inhibit the formation of oxygen species than SC-CO_2_ extracts.

On the other hand, in order to obtain extensive knowledge of the phenolic composition of *E. globulus* and *S. officinalis* PLE-NaDES extracts, characterization by HPLC-QTOF-MS was performed. Compounds were identified by observing their fragmentation and comparing the results with the FOODB database and data from the literature. Table 3 shows the identification of a total of 23 phenolic compounds in *E. globulus*. In general, the HPLC-QTOF-MS analysis revealed that this plant is mainly composed of flavonoids and hydrolyzable tannins. In particular, different flavonoids were identified in high abundance. Quercetin-glucuronide (number 16, Figure S3A) was highlighted as the most abundant flavonoid identified in *E. eucalyptus* PLE-NaDES extracts, with an *m*/*z* ion at 477 [M-H] from which the fragmentation of the glucuronide fraction occurs, resulting in the aglycone quercetin ion at *m*/*z* 301 [32]. After quercetin-glucuronide, quercetin-galactoside-gallate (number 14, Figure S3B), followed by catechin (number 6), was the identified compound in *E. eucalyptus* PLE-NaDES extracts with the highest abundance. In addition, gallic acid (number 1, Figure S3C) was identified with an *m*/*z* ion at 169 [M-H], along with its characteristic fragment ions at 121 [32]. It has been reported by several authors that *E. globulus* is rich in ellagic acid; however, the HPLC-QTOF-MS analysis did not show a clear fragmentation pattern of this compound [32,33,34].

Additionally, as can be observed in Table 3, the majority of identified phenolic compounds in *E. globulus* PLE-NaDES extracts crossed the BBB. In fact, permeable compounds accounted for 70% of the abundance in the HPLC-QTOF-MS analysis compared to the initial PLE-NaDES extract, except for gallic acid, HHDP galloylglucose, digalloylglucose, isorhamnetin-hexoside, methyl ellagic acid-pentoside, and isorhamnetin-rhamnoside, which presented lower abundance values. In particular, pedunculagin was identified with an *m*/*z* ion and 783 [M-H], along with corresponding fragment ions at 481, which originated from the loss of a deprotonated HHDP glucose molecule, and at 300 from the loss of HHDP. Only 5% of pedunculagin crossed the BBB. In addition, chlorogenic acid (number 9), tellimagrandin I (number 10), naringenin (number 22), and cypellocarpin C (number 23) were unable to cross the barrier. However, non-permeable compounds were not detected in the non-permeable fraction as they were probably retained in the BBB. An exception was observed for protocatechuic acid (number 3), dimethyl-hesperetin (number 21), and naringenin (number 22), where 22, 8, and 43% of their abundances, respectively, were detected in the non-permeable fraction.

Regarding *S. officinalis*, a total of 25 phenolic compounds were identified in the PLE-NaDES extract, highlighting the family class of hydroxycinnamic acids with the highest number of different phenolic compounds identified (10 compounds) (Table 4). In particular, a characteristic hydroxycinnamic acid that is frequently found in *S. officinalis* was identified as salvianic acid C (number 9), with an *m*/*z* ion at 377 [M-H]^−^ and corresponding fragments at 179 [M-H-C_9_H_8_O_4_]^−^, 161 [M-H-C_9_H_8_O_4_-OH]^−^, and 135 [M-H-C_9_H_8_O_4_-OH-H_2_O]^−^ [35]. In addition, rosmarinic acid was identified as the major hydroxycinnamic acid in this extract, with an *m*/*z* ion at 359 [M-H]^−^ that exhibited 197, 179, 161, 151, and 133 fragment ions from the cleavage of the ester bond, loss of hydroxyl groups, and breakdown of the aromatic ring [36] (Figure S4A). However, the most representative compounds, taking into account their abundance in the HPLC-QTOF-MS analysis, were phenolic diterpenes. Carnosol (number 23, Figure S4B) was the compound identified with the highest abundance in the PLE-NaDES extract from *S. officinalis*. This compound was detected with an *m*/*z* ion at 329 [M-H] and its characteristic fragment ion at 285 [M-H-CH_3_-C_2_H_3_O]^−^ [35]. After carnosol, carnosic acid (number 24, Figure S4C) and methyl carnosate (number 25, Figure S4D) were the major compounds detected in this plant.

Despite phenolic diterpenes being the majority compounds in *S. officinalis*, these compounds were not permeable since they were unable to cross the BBB. A minimum abundance percentage of carnosic acid (0.8%) and methyl carnosate (1.6%) crossed the BBB. Although 21% of the carnosol abundance of the PLE-NaDES extract crossed the barrier, this percentage was relatively low compared to the other permeable compounds. Similarly, caffeic acid (number 6) scarcely crossed the BBB (10%), while around 50% of *p*-coumaric acid (number 7), ethyl caffeate (number 17), epirosmanol (number 20), and rosmadial (number 22) crossed the barrier. In particular, seven phenolic compounds were not permeable, including quinic acid (number 4), caffeoylquinic acid (number 5), lithospermic acid (number 10), rosmarinic acid glucoside (number 12), isorhamnetin-hexoside (number 13), apigenin-rutinoside (number 14), and dimethylquercetin (number 21). The rest of the identified compounds totally crossed the BBB, except for danshensu (number 1) and quercetin glucuronide (number 11), which presented permeability percentages of 72 and 81%, respectively.

### 2.3. Relationship Between Individual Phenolic Compounds and Terpenoids, with the Antioxidant and Anticholinergic Capacities of SC-CO_2_ and PLE-NaDES Extracts from E. globulus and S. officinalis

A multivariate statistical analysis was conducted to evaluate the impact of the shared terpenoid and phenolic composition of SC-CO_2_ and PLE-NaDES extracts from *E. globulus* and *S. officinalis* on their biological activities and to compare both plants.

**Figure 3:**
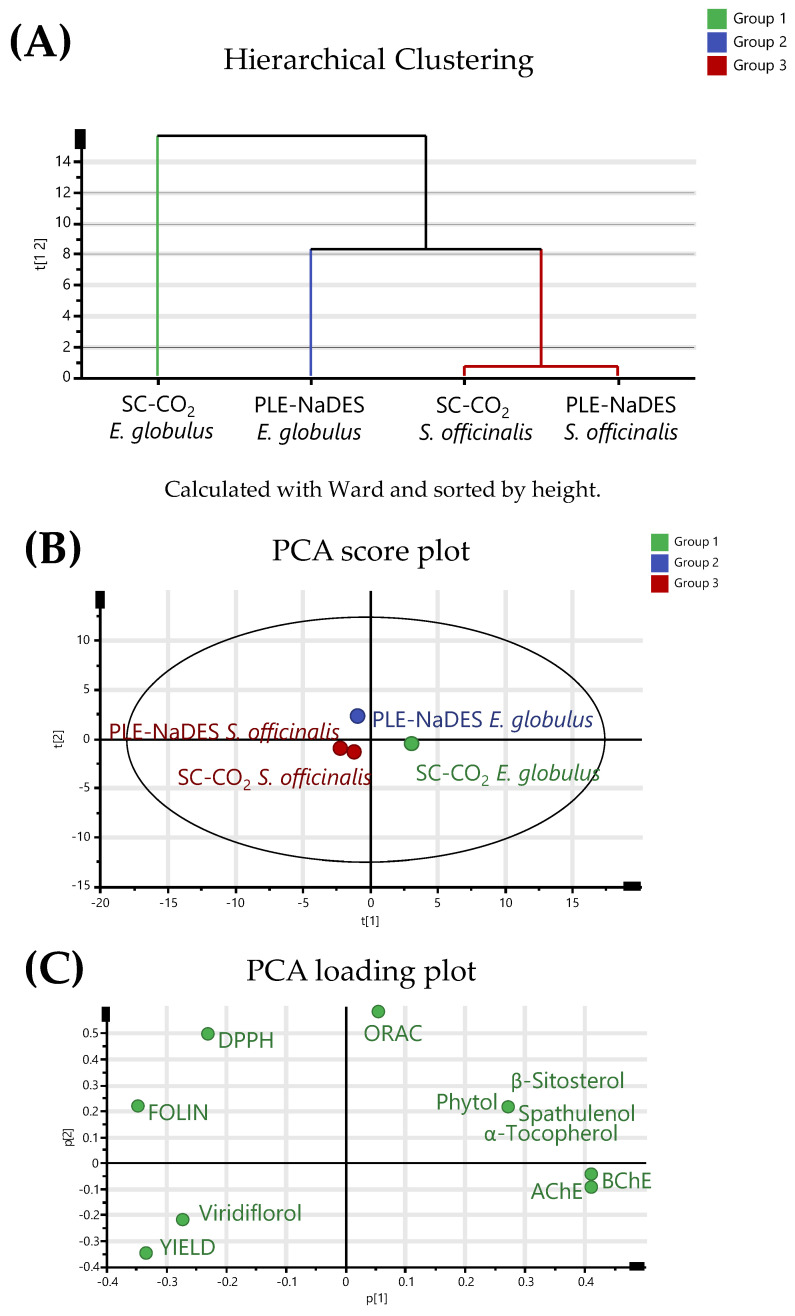
(**A**) Dendrogram generated through HCA using the Ward method, organized by the extraction yield, TPC, and antioxidant and anticholinergic properties of SC-CO_2_ and PLE-NaDES extracts from *E. globulus* and *S. officinalis*. (**B**) Score plot derived from the PCA of the various extraction methods, showing correlations with the extraction yield, TPC, antioxidant activity, and anticholinergic properties of the analyzed plants. (**C**) Loading plot obtained from PCA.

Hierarchical cluster analysis (HCA) was applied to categorize plant extracts into groups based on their phenolic content, terpenoids, total phenolic content (TPC), antioxidant activity, and anticholinergic properties, resulting in three distinct clusters (Figure 3A). One cluster grouped the SC-CO_2_ extract obtained from *E. globulus* (green type), another the PLE-NaDES extract from *E. globulus* (blue type), while the third cluster grouped both extracts (SC-CO_2_ and PLE-NaDES) from *S. officinalis* (red type). Additionally, PCA allows for identifying the most significant variables (principal components) and differentiating the extracts based on their terpenoid and phenolic contents, as well as their biological activities. Two principal components described 93.17% of the total variability. As can be observed in Figure 3B, PCA grouped the extracts similarly to HCA (using the same colors). Extracts belonging to each group exhibited similar terpenoid and phenolic compositions, as well as comparable biological capacity. For instance, as shown by the overlap in Figure 3B,C, the red group that corresponded to *S. officinalis* extracts presented the highest antioxidant capacity, as determined by the ORAC assay, and the highest anticholinergic capacity, as evaluated by the AChE and BChE methods, because this group was opposite to these capacities expressed as IC_50_ values in the PCA loading plot (Figure 3C). Moreover, the SC-CO_2_ extract from *S. officinalis* exhibited the highest viridiflorol content, along with the corresponding PLE-NaDES extract, due to being in the same quadrant in the PCA score plot. Both extracts exhibited higher antioxidant capacities, as determined by the ORAC assay, as they are located in the opposite quadrant to these extracts in the PLCA loading plots according to their IC50 values. This means that the extraction yield and viridiflorol content in both extracts positively influenced the antioxidant capacity determined by the ORAC assay. In contrast, the PLE-NaDES extract from *E. globulus* presented a higher antioxidant capacity as determined by the DPPH assay; thus, this extract was separated from the red group. On the other hand, despite the green group (SC-CO_2_ extract from *E. globulus*) being the richest in tocopherols, spathulenol, and phytol, it exhibited the lowest bioactivity because this group was in the same quadrant as these capacities expressed as IC_50_ values in the PCA loading plot.

## 3. Discussion

### 3.1. Elucidation of the Bioactive and Chemical Profiles of Non-Polar Compounds from E. globulus and S. officinalis SC-CO_2_ Extracts

*E. globulus* and *S. officinalis* plants are characterized by their bioactive properties attributed to their composition in essential oils and phenolic compounds. These compounds obtained from *E. globulus* and *S. officinalis* have presented interesting antioxidant, antibacterial, and neuroprotective properties [37,38,39]. However, despite some authors observing that these plants possess neuroprotective compounds that could be used for pharmaceutical purposes to prevent the development of neurodegenerative disorders such as AD, their extraction, bioactive determination, characterization, and BBB permeability have been scarcely studied. For this reason, the elucidation of the neuroprotective and antioxidant capacities of compounds with different chemical properties obtained from both plants was carried out. In this sense, a sequential extraction process that consisted of SC-CO_2_ extraction to obtain the most non-polar compounds (terpenoids) followed by a PLE-NaDES extraction process to recover compounds with medium polarity, such as phenolic compounds, was performed on both plants. In addition, the BBB permeability of the obtained compounds was studied.

First, the SC-CO_2_ extraction method applied consisted of the use of CO_2_ as the extraction solvent (without a co-solvent) at a low temperature (60 °C) and pressure (200 bar). This prevents the degradation of thermolabile compounds and the co-extraction of compounds with medium polarity. As can be observed in Figure 1, the extraction yields of *E. globulus* and *S. officinalis* extracts were slightly lower than those obtained by other authors [40,41]. In particular, Rodrigues et al. [40] achieved similar extraction yields (1.52%) from *E. globulus* to those in our study (1.22%) by employing the same extraction pressure at 40 °C for six hours. In addition, Valentina Pavic’ et al. [41] demonstrated that higher extraction pressures and temperatures imply higher extraction yields in *S. officinalis*. In addition, the higher extraction yields observed by other authors are because the majority of them employed a co-solvent during the extraction process, particularly ethanol (at different amounts such as 2.5, 5, and 7.5%), along with high extraction pressures (between 172 and 300 bar) [42,43]. In fact, Rodrigues et al. [40] reported that the use of 250 bar with 5% ethanol as a co-solvent provided higher extraction yields (3.95%) from *E. globulus* than the use of 200 bar without a co-solvent (1.52%). The addition of a co-solvent implies the extraction of both polar and non-polar compounds, increasing the extraction yields.

Nevertheless, no co-solvent was used in this study, allowing for the extraction of a purer fraction of non-polar compounds. This approach was designed to facilitate a biorefinery process aimed at obtaining distinct fractions for further applications. Thus, despite obtaining lower extraction yields, a temperature of 60 °C and 200 bar of pressure were applied according to Domínguez-Rodríguez et al. (2024) [27].

Concerning the bioactivity of these non-polar compounds obtained by SC-CO_2_, *S. officinalis* presented higher antioxidant and anticholinergic capacities than *E. globulus (*Figure 1*)*. *S. officinalis* DPPH values were in line with the results of Branimir Pavlić et al. [44], where traditional extraction techniques, such as hydrodistillation and Soxhlet extraction, and modern extraction techniques, such as SC-CO_2_, were compared. In particular, it was observed that the SC-CO_2_ process under a higher extraction pressure (300 bar) and the same extraction temperature as this study provided extracts with higher DPPH values (0.987 µmol Trolox/g extract), while lower extraction pressures (100 bar) indicated lower extraction efficiencies of antioxidant non-polar compounds. This indicated that higher extraction pressures enhance the recovery of non-polar compounds; however, a higher content of phenolic compounds could be co-extracted, as can be observed in our study.

On the other hand, data from the literature on the anticholinergic properties of *E. globulus* and *S. officinalis* are very limited. Smail Aazza et al. [45] showed that the essential oil obtained by steam distillation from *E. globulus* showed a higher anticholinergic capacity, as evaluated by the AChE assay (IC_50_ value of 129.8 µg/mL), than *Citrus aurantium L., Cupressus sempervirens L Foeniculum vulgare Mill.*, *and Thymus vulgaris L.* plants, as well as compared to our SC-CO_2_ *E. globulus* extract. However, steam distillation requires high temperatures and pressures, leading to increased energy use and potential greenhouse gas emissions. In contrast, SC-CO_2_ is more energy-efficient and generates less environmental waste. While steam distillation may be effective in certain cases, SC-CO_2_ is generally more sustainable due to its lower energy demands and cleaner process [46]. In agreement with our study, it has been observed that AChE enzymes have a higher inhibition capacity than BChE enzymes in *S. officinalis* extracts obtained by SC-CO_2_ [47]. An extract may show greater inhibition of AChE than BChE due to structural and functional differences between the enzymes. AChE’s active site is more specific to acetylcholine, which may make it more susceptible to compounds in the extract that mimic or interfere with acetylcholine activity. Additionally, differences in peripheral binding sites and substrate preferences can enhance the extract’s affinity for AChE. The chemical composition of the extract likely aligns better with AChE’s binding characteristics, resulting in stronger inhibition compared to BChE. In particular, Chen et al. [47] presented SC-CO_2_ *S. officinalis* extracts achieved under unspecified conditions with a higher BChE inhibition capacity than our SC-CO_2_ *S. officinalis* extracts. This could be due to differences in extraction conditions, as well as the edaphoclimatic conditions of the plant collection site, which influence the composition of the extracts and their bioactivity.

In addition, different terpenoid compositions were seen in the GC–MS analysis between *E. globulus* and *S. officinalis* SC-CO_2_ extracts (Table 1 and Table 2). However, it was observed that SC-CO_2_ extracts from *S. officinalis* exhibited higher total peak areas of identified terpenoids than SC-CO_2_ *E. globulus* extracts. This is probably because the high bioactivity of the SC-CO_2_ *S. officinalis* extract was due to the high terpenoid content. Despite this, the multivariate statistical analysis (Figure 3) showed that the content of non-polar compounds identified in both plants was superior in the *E. globulus* extract, and it was noted that these compounds do not provide antioxidant or anticholinergic capacities to the extract like those compounds that were identified in high amounts in *S. officinalis* but not in *E. globulus*. In particular, Perry et al. [38] observed that the anticholinergic capacity of terpenoids of *S. officinalis* results from a synergistic effect of the components. In fact, the anticholinergic capacity of individual terpenoids has been tested and shown to be ineffective.

Terpenoids identified in SC-CO_2_ *E. globulus* extracts in this study were also detected by several authors in other *E. globulus* extracts, such as cineole, camphene, terpineol, spathulenol, and aromadendrene epoxide [18,48]. Different terpenoid concentrations were observed in *E. globulus* among studies; however, sesquiterpenoid is the main family of terpenoids detected in this plant, regardless of the area where the plant was collected and the extraction technique used [18]. In fact, Singh et al. [48] exhibited *E. globulus* SC-CO_2_ extracts rich in sesquiterpenoids, particularly in α-selinene, while sphatulenol was the most prevalent sesquiterpenoid identified in our study in this plant. An interesting anticholinergic capacity of sesquiterpenes has been observed in plants by several authors [5]. In particular, caryophyllene-type terpenoids that were detected in our *E. globulus* SC-CO_2_ extract have been reported for their potent AChE inhibitory effect. Zardi-Bergaoui et al. [49] indicated that the AChE inhibitory capacity of these compounds depends on the substitution positions and configuration of stereogenic centers within the caryophyllene basic skeleton. In addition, spathulenol has shown a great neuroprotective capacity by restoring abnormal cellular conditions induced by neuronal damage through treatment with 5-hydroxydopamine [50]. This means that the obtained SC-CO_2_ *E. globulus* extracts could be an interesting natural source of spathulenol and other bioactive compounds for promising potential therapies for the treatment of neurodegenerative diseases such as AD.

Regarding *S. officinalis*, a similar terpenoid profile to this study was observed in the literature data, with concentrations varying depending on the extraction technique and extraction conditions used [51,52,53]. Coinciding with our study, Aleksovski et al. [52] showed that manool was the most abundant compound in *S. officinalis* extracts obtained by SC-CO_2_ at 128 bar and 50 °C. In addition, hinokione was one of the most abundant terpenoids in the SC-CO_2_ extract obtained in this study from *S. officinalis*. The manool content in *S. officinalis* has been positively correlated with the antioxidant capacity of the extracts [54]. In addition, hinokione has been characterized by its hypoglycemic effects on experimental animal models [55]. Although the neuroprotective capacity of both compounds has not yet been investigated separately, Perry et al. [38] demonstrated that the anticholinergic activity of species in the *Salvia* genus is due to a synergistic effect among terpenoid constituents such as cineole, pinene, limonene, camphor, and caryophyllene, which were also identified in our study.

Assessing the permeability of bioactive molecules across the BBB is a crucial step in screening neuroprotective compounds with the potential to reach the central nervous system (CNS) to exert their effect. The parallel artificial membrane permeability assay (PAMPA) is a non-cellular in vitro test that can accurately simulate passive permeability through the BBB, although it does not consider active transport (uptake and efflux) and the presence of metabolizing enzymes that together regulate the passage of specific molecules from the bloodstream to the CNS and vice versa [56].

In this sense, in order to determine the bioavailability of anticholinergic non-polar compounds identified in SC-CO_2_ extracts, the PAMPA was performed. As can be observed in Table 1 and Table 2, few terpenoids were able to cross the BBB. A higher content and number of terpenoids from *E. globulus* crossed the BBB compared to *S. officinalis* extracts. In particular, among the permeable caryophyllene, globulol, aromadendrin, acetoxykauranal, viridiflorol, and guaiol compounds from *E. globulus* extracts, caryophyllene has shown interesting neuroprotection activities by decreasing neuroinflammation and inhibiting necroptotic cell death [57,58]. The neuroprotective properties of globulol make it a candidate for therapeutic applications for AD and other age-related neurodegenerative disorders. Studies have demonstrated that essential oils rich in globulol can improve cognitive function and reduce β-amyloid-induced toxicity in animal models [59]. Moreover, it has been observed that viridiflorol can activate the phosphoinositide 3-kinase (PI3K)/Akt signaling pathway, which plays a crucial role in cell survival and apoptosis prevention. This pathway is essential for promoting neuroprotection against amyloid-beta-induced toxicity, a significant factor in AD [60].

On the other hand, only three compounds from *S. officinalis* extracts crossed the BBB, namely camphene, totarol, and hinokione (Table 2). Among them, totarol is characterized by its protective effect against cerebellar granule neurons and cerebral cortical neurons from damage caused by glutamate-induced injury or oxygen and glucose deprivation [61]. This means that both plant extracts could be interesting sources of bioavailable terpenoids with neuroprotective effects.

### 3.2. Bioactive and Chemical Profiles of Polar Compounds from E. globulus and S. officinalis PLE-NaDES Extracts

Soft SC-CO_2_ extraction conditions (200 bar at 60 °C) were applied in order to obtain an extract that was as pure as possible for non-polar compounds, mainly terpenes, avoiding the degradation of thermolabile compounds. This means that the residue of SC-CO_2_ could retain interesting polar bioactive compounds, such as polyphenols. In fact, Domínguez-Rodríguez et al. [27] demonstrated that, under these extraction conditions, a large amount of phenolic compounds with antioxidant and anticholinergic capacities can be recovered from the residue of *C. reticulata* leaves. In particular, these authors optimized a sustainable extraction method employing PLE combined with NaDES by testing different NaDES. It was observed that NaDES composed of ChCl:Gly (1:2) allowed the authors to obtain the extracts with the highest bioactive phenolic compounds compared to other NaDES and with the use of conventional solvents such as ethanol/water (70;30, *v*/*v*). Different authors indicated that the high extraction efficiency of ChCl:Gly (1:2) on the recovery of phenolic compounds could be due to the strong interaction formed between ChCl and Gly with phenolic compounds, as well as high polarity and diffusivity, which increases their release from the matrix. In addition, the pH of these NaDES (around 2) can facilitate the hydrolysis of the cell wall, releasing phenolic compounds from the extraction medium [23,62,63]. Additionally, the use of NaDES not only enhances the extraction yields of phenolic compounds but also provides a protective effect against their degradation under adverse temperature and oxygen conditions, offering an innovative approach for preserving bioactive compounds [64]. Thus, and considering the extraction efficiencies of PLE using the NaDES reported by several authors, the optimized PLE-NaDES extraction conditions (with ChCl:Gly and using 57.9% water) achieved by Domínguez-Rodríguez et al. [64] were applied to extract phenolic compounds from the residue of SC-CO_2_ of *E. globulus* and *S. officinalis*. This process was designed to maximize the exploitation of the matrix, yielding sustainable extracts with diverse compositions for various applications, thereby contributing to the circular economy. As a biorefinery approach, it employs environmentally sustainable extraction methods, ensuring alignment with ecological principles.

As can be observed in Figure 2, PLE-NaDES extracts from *S. offiicinalis* resulted in higher extraction yields, TPC values, and antioxidant and anticholinergic capacities than *E. globulus*. Several authors showed that the *S. offiinalis* plant is one of the most used medicinal plants for pharmaceutical purposes due to its high antioxidant polyphenol content, which is even higher than *E. globulus* [65,66,67]. In addition, to the best of our knowledge, there are no studies in which PLE has been applied to extract phenolic compounds from *E. globulus*. In particular, Gullón et al. [68] compared different advanced extraction techniques for the recovery of phenolic compounds from *E. globulus* with conventional extraction. These authors observed that advanced extraction techniques, particularly MAE, provided higher extraction efficiencies with lower energy consumption compared to conventional extraction. Ethanolic MAE extracts from *E. globulus* have presented higher antioxidant phenolic contents than when using DES under maceration. However, there are no comparative studies on the inclusion of NaDES or DES into advanced extraction techniques such as PLE for the extraction of phenolic compounds from this plant. Most likely, this combination enhances the recovery of bioactive compounds compared with the use of advanced extraction techniques separately from the NaDES, as Domínguez-Rodríguez et al. [27,64] and Oliveira et al. [23] observed. The antioxidant capacity of *E. globulus* has been related to the presence of phenolic acids, catechin, and flavonoids. In particular, the flavonoid content of this plant has been positively correlated with its antioxidant capacity [69]. In agreement with the literature data, catechin was one of the main phenolic compounds identified in *E. globulus* extracts. However, other compounds were found in higher amounts in this study, such as quercetin-glucuronide or galactoside, while other authors reported other compounds in higher amounts, such as digalloylglucose or isorhamnetin-rhamnoside, among others [69,70]. Differences in phenolic composition among studies could be due to the type of extraction technique used, as well as the climatic conditions of the place where the plant was collected, which determine its phenolic content. The high content of quercetin-galactoside and glucuronide was noted, and these compounds have shown interesting therapeutic potential for AD and Parkinson’s disease by modulating signaling pathways of neuroinflammation, oxidative stress, and some genes involved in their development [71,72]. In agreement with Nguyen et al. [71], these compounds presented a high BBB permeability (Table 3). These findings highlight the significance of further exploring these environmentally sustainable extracts as valuable sources of natural neuroprotective compounds for use in therapeutic treatments.

On the other hand, phenolic compounds from *S. officinalis* have been widely recovered by conventional extraction techniques; however, some researchers have focused their efforts on the development of more sustainable extraction methodologies, for example, by using PLE [73,74,75]. In particular, PLE of phenolic compounds from this plant has been carried out by employing aqueous organic solvents such as ethanol and methanol, resulting in richer extracts compared to conventional extraction techniques [73,74]. The results on TPC and bioactivity in the literature data cannot be compared with our results due to differences in their expression. In addition, there are no studies on PLE combined with NaDES for the recovery of phenolic compounds from *S. officinalis*. In agreement with the literature, this plant is characterized by its phenolic diterpene contents, particularly carnosol, carnosic acid, and methyl carnosate (Table 4). These compounds have been related to the antioxidant effect of the plant by several authors [74,76]. However, these compounds did not show a high BBB permeability in this study. In addition to phenolic diterpenes, rosmarinic acid was also one of the most abundant phenolic compounds in our extracts. This compound is a well-recognized natural antioxidant found in a variety of plants. Its antioxidant potential has been demonstrated to be over three times greater than Trolox. In addition, it inhibits xanthine oxidase and is likely to neutralize excess free radicals in the body. Furthermore, rosmarinic acid has the ability to reduce Mo (VI) to Mo (V), potentially preventing the formation of free radicals triggered by oxidation catalyzed by polyvalent metal ions [75,77]. This compound also reduces the β-amyloid deposition and oxidative stress associated with AD [78,79]. In fact, rosmarinic acid was one of the most BBB-permeable compounds out of the compounds identified in this study to exert a neuroprotective effect. Differences in the permeability of phenolic diterpenes and rosmarinic acid could be due to their molecular size; in fact, phenolic diterpenes have a larger a more complex structure, while rosmarinic acid is smaller and more hydrophilic, enhancing its ability to cross the BBB. In addition to rosmarinic acid, caffeic acid, which has been identified as a major phenolic acid in *S. officinalis* extracts according to the literature, also efficiently crosses the BBB [75,76]. Considering these facts, it can be suggested that PLE-NaDES extraction could present a new, sustainable alternative for the efficient recovery of neuroprotective compounds from *S. officinalis*.

## 4. Materials and Methods

### 4.1. Chemical and Reagents

HPLC-grade solvents (ethanol, methanol, and ethyl acetate), as well as formic acid, were provided from VWR Chemicals (Barcelona, Spain), while LC–MS-grade acetonitrile and water were acquired from LabScan (Dublin, Ireland). Folin–Ciocalteu reagent was supplied by Merck (Darmstadt, Germany). 4-(amino- sulfonyl)-7-fluoro-2,1,3-benzoxadiazole (ABD-F), 2,2-azobis(2-aminodinopropane) dihydrochloride (AAPH), and galantamine hydrobromide were purchased from TCI Chemicals (Tokyo, Japan). Acetylcholinesterase (AChE) Type VI-S from *Electrophorus electricus*, butyrylcholinesterase (BChE) from equine serum, choline chloride (ChCl), porcine polar brain lipid (PBL), Trizma hydrochloride, fluorescein sodium salt, disodium phosphate, monopotassium phosphate, potassium persulfate, Trolox, sodium carbonate, 1,1-diphenyl-2-picrylhydrazyl (DPPH), ascorbic acid, and gallic acid were provided by Sigma-Aldrich (Madrid, Spain). In addition, glycerol was purchased from Labkem (Barcelona, Spain).

The ultrapure water (18.2 MΩ/cm) was supplied by a Millipore system (Millipore, Billerica, MA, USA).

### 4.2. Samples

The leaves from *E. globulus* and *S. officinalis* were provided by the “Fibreno Officinali” company located in the south of Italy (Isola del Liri, Lazio). The plants’ leaves were harvested in October 2024. The leaves were freeze-dried in a Buchi Lyovapor 1–200 for three days at 0.200 mbar and −55 °C. After, leaves were ground using a commercial blender and stored at −80 °C until further analysis.

### 4.3. Supercritical CO_2_ Extraction of Non-Polar Compounds

The extraction of terpenoids was carried out with a compressed fluid extractor composed of a CO_2_ pump (PU-2080 Plus CO2; Jasco, Hachioji, Japan), an oven, and a manual micrometering needle valve (Vici-Valco Instruments Co., Inc., Houston, TX, USA). The extraction conditions were set to 200 bar of pressure and 60 °C of temperature for 2 h of time, according to the optimized extraction conditions used to obtain terpenoids from citrus leaves achieved by Domínguez-Rodríguez et al. [27]. Briefly, 3 g of dried leaves were mixed with 7.5 g of glass balls in a 34 mL extraction cell. Pure CO_2_ was used as a solvent with a flow rate of 4 mL/min. The extracts were then dissolved in EtOH 100%, evaporated with N_2_ stream, and stored at −20 °C until their analysis. All the extractions were performed in triplicate. The SC-CO_2_ residue was stored for the subsequent extraction of phenolic compounds by PLE-NaDES.

### 4.4. Pressurized NaDES Extraction

Phenolic compounds were obtained from the SC-CO_2_ residue of *E. globulus* and *S. officinalis* leaves through PLE combined with NaDES composed of Choline Chloride:Glycerol in a 1:2 molar ratio with 57.9% water at 64 °C for 27 min and 1500 psi, according to Domínguez-Rodríguez et al. [27] for the extraction of phenolic compounds from citrus leaves. The extractions were carried out in a Dionex Accelerated Solvent Extraction (ASE) 200 (Sunnyvale, CA, USA) with 5 mL extraction cells, containing 0.8 g SC-CO_2_ residue of *E. globulus* leaves mixed with 2 g of sea sand, while 0.4 g SC-CO_2_ residue of *S. officinalis* were mixed with 1 g of sea sand.

NaDES were synthesized according to the method of Hernández-Corroto et al. [80], by mixing both components with 57.9% water at 80 °C for 1 h. The extractions were performed in triplicate.

### 4.5. Solid-Phase Extraction to Remove NaDES from the Extracts

A solid-phase extraction (SPE) process following the protocol of Silva et al. [81] was applied as a purification process to remove NaDES from PLE-NaDES extracts to avoid interferences with spectrophotometric assays. For that, C-18 cartridges (Supelclean LC-18 SPE, 500 mg, EEUU) were placed in a vacuum system and conditioned with 2.5 mL of MeOH 100% followed by 7.5 mL of 0.35% acidified water with formic acid. Subsequently, 2.5 mL of PLE-NaDES extract was loaded in the cartridge, and 2.5 mL of 0.35% acidified water with formic acid was added to elute NaDES. Finally, phenolic compounds were recovered for the cartridge by eluting 5 mL of EtAC 100% followed by 5 mL of a 0.1% acidified methanol with formic acid.

The extracts were evaporated with N_2_ stream and stored at −20 °C until their analysis. For the analysis, the dried extracts were solubilized in 50% ethanol.

### 4.6. Total Phenolic Content (TPC)

The total phenolic content (TPC) of SC-CO_2_ and PLE-NaDES extracts from *E. globulus* and *S. officinalis* leaves was determined using the Folin–Ciocalteu (FC) assay based on the protocol described by M. Koşar et al. [82]. The absorbance was measured at 760 nm using a BioTek Synergy HT UV–Vis spectrophotometer microplate reader (BioTek Instruments, Winooski, VT, USA). Results were represented as mg gallic acid equivalents (GAE)/g extract, employing a calibration curve with gallic acid.

### 4.7. Anticholinergic Activity Determination

The anticholinergic activity of extracts was measured by the inhibition of acetylcholinesterase (AChE) and butyrylcholinesterase (BChE) enzymes following the method of Sánchez-Martínez et al. [7]. The results were expressed as IC_50_ (µg/mL extract). Anticholinergic capacity tests were carried out in a spectrophotometer microplate reader (Cytation 5 imaging reader with auto-disperser, BioTek Instruments, Winooski, VT, USA).

### 4.8. Antioxidant Capacity Determination

The antioxidant capacity was evaluated by the interaction between extracts and the 1,1-diphenyl-2-picrylhydrazyl (DPPH) free radical according to W. Brand-Williams et al. [83]. The absorbance was measured at 516 nm using a BioTek Synergy HT UV–Vis spectrophotometer microplate reader (BioTek Instruments, Winooski, VT, USA). Results were expressed as µmol Trolox equivalents/g extract. Trolox equivalent values were achieved across four different concentrations of each extract, showing a linear response ranging from 20% to 80% compared to the initial absorbance.

In addition, the antioxidant capacity of the extracts was also determined using the ORAC method, following the protocol of Boxin Ou et al. [84]. Fluorescence was determined (λexcitation = 485 nm; λemission = 530 nm) every 5 min at 37 ◦C for 1 h in a BioTek Synergy HT UV–Vis spectrophotometer microplate reader (BioTek Instruments, Winooski, VT, USA). The results were expressed as mg ascorbic acid/g extract using ascorbic acid as the standard.

### 4.9. Gas Chromatography–Mass Spectrometry (GC–MS) Analysis

Terpenoids from *E. globulus* and *S. officinalis* leaf extracts obtained by SC-CO_2_ were analyzed employing a Shimadzu GCMS-QP2010 SE Single Quadrupole GC–MS. The separation was carried out using an Agilent Zorbax DB5-MS + 10 m Duraguard Capillary Column (30 m × 0.25 mm × 0.25 µm) with helium gas as the carrier at a linear velocity of 32.5 cm/s. The oven temperature was optimized for each medicinal plant. For the analysis of SC-CO_2_ *E. globulus* extracts, the oven temperature began at 45 °C and was raised to 200 °C at 10 °C/min, followed by an increase of 5 °C/min up to 300 °C. Then, the temperature was increased to 325 °C at 2 °C and maintained for 2 min. Concerning SC-CO_2_ *S. officinalis* extracts, the oven temperature started at 45 °C and was raised to 200 °C at 10 °C/min, followed by an increase to 300 °C at 2 °C/min. Finally, the temperature was increased up to 325 °C at 2 °C/min and maintained for 2 min.

The injection volume was 1 µL in split mode using an injection temperature of 250 °C. The mass analyzer was set to SCAN mode, with a scan speed of 1428 amu/s, covering a mass range from *m*/*z* 50 to 550 and an event time of 0.40 s. An ion source temperature of 250 °C was used with an interface temperature of 335 °C.

Shimadzu GC Solution software (Ver. 2.32) was used for data processing, employing the commercial Wiley and Nist mass spectral database.

### 4.10. HPLC-DAD-QTOF-MS Analysis

Phenolic compounds obtained from the SC-CO_2_ residue of *E. globulus* and *S. officinalis* leaves by PLE-NaDES were characterized using an Agilent LC system 1100 (Agilent Technologies, Palo Alto, CA, USA) with a diode array detector (DAD) and a quadrupole time-of-flight mass spectrometer (QTOF-MS) featuring an orthogonal electrospray ionization (ESI) source. An Agilent ZORBAX Eclipse Plus C18 analytical column (100 × 2.1 mm, 1.8 µm particle size) with a ZORBAX Eclipse Plus C18 Guard 3PK column (5 × 2.1 mm) was used for chromatographic separation, following the method of Sánchez-Martínez et al. (2022) [85]. Briefly, mobile phases consisting of (A) water with 0.1% of formic acid and (B) acetonitrile with 0.1% of formic acid were used in a gradient elution as follows: 0 to 30% B (0–7 min); 30 to 80% B (7–9 min); 80–100% B (9–11 min); 100% B (11–13 min); 100 to 0% B (13–14 min). An injection volume of 2 µL, a flow rate of 0.5 mL/min, and a column temperature of 40 °C were applied. The mass spectrometer operated in negative ion mode, scanning a mass range of *m*/*z* 50 to 1400. The capillary voltage, nebulizer pressure, drying gas flow rate, and gas temperature were set at 3000 V, 40 psig, 8 L/min, and 300 °C, respectively. The fragmentor voltage (cone voltage after the capillary) was adjusted to 110 V, while the skimmer and octapole voltages were 45 V and 750 V, respectively. The source sheath gas temperature was maintained at 350 °C with a flow rate of 11 L/min. Each extract was analyzed in duplicate.

### 4.11. Parallel Artificial Membrane Permeability Assay for the Blood–Brain Barrier (PAMPA-BBB)

The PAMPA-BBB experiment was conducted following the method described by Könczöl et al. [86]. Briefly, the BBB solution was achieved by mixing 8 mg of PBL and 4 mg of cholesterol dissolved in 600 µL of n-dodecane. Then, 400 µL of buffer (PBS pH 7.4, 10 mM) was mixed with 300 µL of SC-CO_2_ or PLE-NaDES extract at 10 mg/mL to prepare the initial donor solution. The filter membrane of the donor plate was coated with 5 µL of a BBB solution. Next, the acceptor plate was filled with 350 µL of buffer, and the donor plate was placed on top of the acceptor plate. After that, 200 µL of the donor solution stock was added to the donor plate. The assembled plates were covered and incubated at 37 °C for 5 h, away from direct light. After incubation, the plates were separated, and 150 µL of solution from both plates was transferred to a vial and dried to obtain acceptor and donor samples. These dried samples were then reconstituted in 50 µL of EtOH for subsequent GC–MS and HPLC-DAD-QTOF-MS analyses to detect non-polar and polar permeable compounds through the BBB.

### 4.12. Statistical Analysis

Statistical analysis was carried out by applying the Statgraphics Centurion XVII software (Statistical GraphicsCorp, VA, USA) to determine differences among *E. globulus* and *S. officinalis* extracts regarding TPC, antioxidant capacity, and anticholinergic capacity. ANOVA was used to determine statistically significant differences (*p* ≤ 0.05) among the mean values of different extracts at a 95% confidence level. Data were presented as mean ± standard deviation, and all analyses were performed in triplicate for each extract.

## 5. Conclusions

This study is the first to use a combined sequential approach of SC-CO_2_ extraction and PLE-NaDES to obtain bioactive compounds from *S. officinalis* and *E. globulus*, with the aim of obtaining natural extracts with neuroprotective effects by evaluating their BBB permeability.

Comparing the two extraction techniques, PLE-NaDES extracts demonstrated superior antioxidant and anticholinergic capacities compared to SC-CO_2_ extracts. This suggests that polar compounds, particularly those extracted from *S. officinalis*, exhibit greater antioxidant and anticholinergic potential. A total of 21 non-polar compounds were identified in SC-CO_2_ *E. globulus* extracts by GC–MS, mainly sesquiterpenoids, six of which were capable of crossing the BBB. Notably, caryophyllene oxide, globulol, and guaiol permeable compounds have shown interesting neuroprotective capacities, as highlighted by several authors. Concerning SC-CO_2_ *S. officinalis* extracts, 24 non-polar compounds were identified by GC–MS, but only three crossed the BBB. On the other hand, PLE-NaDES extracts from the SC-CO_2_ residues of both plants exhibited a higher BBB permeability for phenolic compounds. Among 25 compounds identified by HPLC-QTOF-MS in PLE-NaDES extracts from both plants, only four from *E. globulus* and seven from *S. officinalis* failed to cross the BBB. Flavonoids from PLE-NaDES *E. globulus* extracts showed remarkable BBB permeability, while rosmarinic acid exhibited particularly high permeability in PLE-NaDES extracts of *S. officinalis*.

The biorefinery process employed yields extracts with distinct compositions: one enriched in terpenoids and another abundant in flavonoids with high neuroprotective capacity and BBB permeability. This strategy facilitates the exhaustive valorization of the plant matrix through sustainable extraction methods, ensuring the maximal recovery of bioactive compounds while contributing to environmental sustainability.

## Figures and Tables

**Table 1:** Compounds identified by GC–MS and their peak areas in the *E. globulus* SC-CO_2_ extracts, as well as in the permeable and non-permeable fractions through the blood–brain barrier (BBB).

ID	Proposed Compound	RT (min)	Molecular Formula	Measured Mass	Main Fragment Ions (*m*/*z*)	SC-CO_2_ Extract *	Permeable BBB Fraction *	Non-Permeable BBB Fraction *
1	Cryptone	9.58	C_9_H_14_O	138	138, 97, 96, 95, 81, 67, 43	743 ± 145	-	293 ± 37
2	Cineole	10.13	C_10_H_18_O	154	126, 111, 58, 43	86 ± 8	-	68 ± 6
3	Limonene dioxide	10.45	C_10_H_16_O_2_	168	153, 107, 55, 43	271 ± 75	-	154 ± 8
4	Camphene	11.06	C_10_H_16_	152	136, 121, 107, 93	415 ± 59	-	149 ± 6
5	Terpineol	12.08	C_10_H_18_O	154	153, 139, 121, 83, 93, 69, 55, 43	573 ± 53	-	57 ± 6
6	Linalool	12.27	C_10_H_18_O	154	122, 121, 93, 83, 69, 67, 55, 43	385 ± 52	-	12 ± 1
7	Terpineol isomer	12.44	C_10_H_18_O	154	111, 93, 71, 69, 57, 55, 43	548 ± 87	-	208 ± 19
8	Piperitone oxide	13.08	C_10_H_16_O_2_	168	139, 125, 97, 69, 55, 43, 41	678 ± 3	-	196 ± 14
9	Spathulenol	14.92	C_15_H_24_O	220	205, 159, 119, 91, 43	9754 ± 731	-	56 ± 3
10	Caryophyllene oxide	16.61	C_15_H_24_O	220	161, 109, 107, 93, 79, 43	3195 ± 268	233 ± 6	880 ± 131
11	Globulol	16.66	C_15_H_26_O	222	204, 161, 109, 93, 69, 55, 43	2182 ± 90	103 ± 6	549 ± 62
12	Aromadendrene epoxide	16.84	C_15_H_24_O	220	149, 147, 121, 107, 105, 95, 91, 55, 43	1482 ± 42	83 ± 6	280 ± 36
13	Acetoxy-kauranal	16.92	C_22_H_34_O_3_	346	159, 147, 135, 131, 109, 107, 55, 43	1242 ± 37	124 ± 6	421 ± 48
14	Patchoulane	17.12	C_15_H_26_	206	149, 107, 105, 91, 79, 67, 55, 41	526 ± 8	-	-
15	Viridiflorol	17.80	C_15_H_26_O	222	204, 189, 149, 135, 109, 95, 93, 81, 71, 59, 43	844 ± 12	125 ± 16	565 ± 70
16	Andrographolide	17.85	C_20_H_30_O_5_	350	187, 159, 145, 133, 107, 105, 91, 79, 77, 67, 55, 43. 41	1171 ± 122	-	116 ± 18
17	Guaiol	18.08	C_15_H_26_O	222	162, 161, 147, 133, 119, 107, 105, 91, 81, 67, 59, 43	792 ± 58	69 ± 18	378 ± 62
18	Bisabolene epoxide	18.96	C_15_H_24_O	220	149, 135, 121, 109, 105, 93, 91, 79, 67, 57, 55, 43	542 ± 9	-	210 ± 38
19	Phytol	21.62	C_20_H_40_O	296	278, 197, 137, 123, 111, 95, 81, 71, 55, 43	14,900 ± 4320	-	3031 ± 956
20	α-Tocopherol	36.08	C_29_H_50_O_2_	430	430, 205, 165, 121, 57, 43	5129 ± 431	-	361 ± 151
21	β-Sitosterol	38.80	C_29_H_50_O	414	414, 396, 329, 303, 255, 231, 163, 145, 119, 105, 81, 55, 43	4736 ± 796	-	706 ± 12

* Peak area expressed as ×10^3^; (-): Not detected.

**Table 2:** Compounds identified by GC-MS and their peak areas in the *S. officinalis* SC-CO_2_ extracts, as well as in the permeable and non-permeable fractions through the blood–brain barrier (BBB).

ID	Proposed Compound	RT (min)	Molecular Formula	Measured Mass	Main Fragment Ions (*m*/*z*)	SC-CO_2_ Extract *	Permeable BBB Fraction *	Non-Permeable BBB Fraction *
1	Pinene	5.58	C_10_H_16_	136	136, 121, 105, 93, 91, 79,77, 67, 53, 41	245 ± 4	-	207 ± 26
2	Borneol	9.28	C_10_H_18_O	154	139, 121, 110, 95, 79, 67, 55, 43, 41	92 ± 54	-	-
3	Citronellol	11.29	C_10_H_20_O	156	138, 123, 109, 96, 95, 81, 69, 55, 44, 41	29 ± 12	-	-
4	Camphene	11.80	C_10_H_16_	152	136, 121, 107, 93	2976 ± 477	158 ± 1	219 ± 57
5	Artemiseole	12.85	C_10_H_16_O	152	137, 109, 95, 91, 79, 77, 67, 57, 55, 43, 41	93 ± 5	-	-
6	Aromadendrene	13.19	C_15_H_24_	204	204, 189, 162, 161, 147, 133, 122, 119, 105, 93, 91, 79, 67, 55, 43, 41	106 ± 43	-	27 ± 7
7	Myrtenol	13.45	C_10_H_16_O	152	121, 119, 108, 96, 91, 82, 79, 77, 67, 55, 53, 43, 41	164 ± 18	-	161 ± 24
8	α-Humulene	13.90	C_15_H_24_	204	204, 161, 121, 119, 115, 107, 105, 103, 95, 93, 91, 81, 79, 77, 67, 65, 55, 53, 43, 40	80 ± 13	-	-
9	Palustrol	14.83	C_15_H_26_O	222	204, 189, 161, 147, 133, 122, 111, 107, 95, 93, 81, 79, 69, 67, 55, 53, 41	1250 ± 150	-	-
10	Spathulenol	14.92	C_15_H_24_O	220	205, 202, 159, 147, 131, 119, 105, 93, 91, 79, 67, 55, 43, 41	407 ± 46	.	-
11	Patchulane	15.01	C_15_H_26_	206	135, 121, 109, 108, 107, 93, 91, 79, 77, 69, 67, 55, 43, 41	277 ± 31	-	-
12	Viridiflorol	15.12	C_15_H_26_O	222	204, 189, 161, 147, 133, 121, 109, 107, 105, 93, 81, 69, 67, 55, 43	5508 ± 397	-	-
13	Ledol	15.25	C_15_H_26_O	222	204, 189, 161, 147, 133, 122, 109, 107, 95, 93, 81, 69, 55, 53, 43	1556 ± 149	-	-
14	Pinene isomer	16.57	C_10_H_16_	136	137, 121, 119, 107, 93, 91, 79, 69, 55, 41	322.5 ± 0.4	-	25 ± 5
15	Germacrene-D	18.89	C_15_H_24_	204	161, 133, 119, 105, 95, 91, 79, 67, 55, 41	508.0 ± 0.1	-	63 ± 13
16	Manool	22.40	C_20_H_34_O	290	272, 257, 204, 189, 177, 161, 148, 137, 121, 109, 107, 95, 81, 71, 69, 55, 43, 41	26,557 ± 11	-	2779 ± 466
17	Phytol	23.26	C_20_H_40_O	296	196, 137, 123, 111, 95, 83, 81, 72, 71, 57, 55, 43, 41	6641 ± 127	-	1792 ± 298
18	Totarol	30.46	C_20_H_28_O_2_	300	300, 285, 257, 243, 229, 217, 217, 128, 115, 83, 69, 55, 43, 41	1824 ± 162	78 ± 7	435 ± 18
19	Carnosol	31.21	C_20_H_26_O_4_	330	287, 286, 271, 243, 215, 204, 187, 143, 128, 115, 91, 77, 55, 43, 41	752 ± 82	-	-
20	Hinokione	42.16	C_20_H_28_O_2_	300	300, 285, 243, 213, 187, 115, 91, 83, 69, 55, 43	21,080 ± 64	69 ± 9	1434 ± 145
21	Ferruginol	44.06	C_20_H_30_O	286	286, 271, 253, 229, 189, 147, 105, 69, 55	4334 ± 82	-	257 ± 35
22	Farnesol	44.51	C_15_H_26_O	222	161, 136, 121, 107, 95, 93, 81, 69, 55, 41	387 ± 8	-	262 ± 32
23	α-Tocopherol	53.87	C_29_H_50_O_2_	430	430, 205, 165, 121, 57, 43, 41	2358 ± 77	-	439 ± 48
24	β-Sitosterol	59.00	C_29_H_50_O	414	414, 396, 329, 303, 255, 213, 173, 159, 145, 133, 131, 119, 109, 105, 95, 91, 81, 69, 67, 57, 55, 43, 41	2905 ± 27	-	2152 ± 267

* Peak area expressed as ×10^3^; (-): Not detected.

**Table 3:** Phenolic compounds identified from PLE-NaDES extracts of *E. globulus* with their respective abundances obtained in the HPLC-QTOF-MS analysis, and permeable and non-permeable compounds through the BBB from the PLE-NaDES extract.

ID	RT (min)	Proposed Compound	*m*/*z* [M-H]^−^	Main Fragment Ions	PLE-NaDES *	Permeable Fraction *	Non-Permeable Fraction *
1	1.568	Gallic acid	169.0151	125.0236, 108.0207, 79.0183, 69.0336, 51.0231	142 ± 9	85 ± 16	-
2	2.798	HHDP galloylglucose	633.0768	301.0002, 275.0217, 249.0374, 231.7478	15 ± 4	10 ± 1	-
3	2.836	Protocatechuic acid	153.0200	109.0285, 108.0213	14 ± 2	13 ± 1	3.3 ± 0.6
4	2.953	Pedunculagin	783.0736	481.0623, 300.9975, 275.0200	19 ± 2	1.1 ± 0.7	-
5	3.029	Methyl gallate	183.0307	168.0088, 156.0095, 138.9440, 124.0158, 101.6277, 87.2672, 78.0102	21 ± 2	19 ± 5	-
6	3.264	Catechin	289.0741	246.0853, 221.0821, 203.0697, 175.0747, 149.0233, 125.0238, 109.0301, 89.0242, 71.0126, 59.0145	105 ± 4	98 ± 13	-
7	3.338	Digalloylglucose	483.0815	331.0669, 313.0553, 271.0458	60 ± 6	25 ±11	-
8	3.409	Digalloylglucose isomer	483.0815	313.0531, 271.0477, 211.0242, 169.0139, 151.0027, 124.0151	54 ± 15	56 ± 4	-
9	3.459	Chlorogenic acid	353.0904	191.0565, 179.0358, 173.0455, 161.0615, 121.8473	17 ± 1	-	-
10	3.815	Tellimagrandin	785.0890	633.0647, 615.0651, 483.0839, 419.0577, 300.9995, 275.0198	45 ± 5	-	-
11	4.089	Trigalloylglucose	635.0933	483.0803, 465.0670, 313.0550	21 ± 4	22.3 ± 0.9	-
12	4.857	Tetragalloylglucose	787.1044	635.0847, 617.0757, 465.0603, 169.0132	14 ± 2	18 ± 1	-
13	4.981	Methylphloroglucinol-digalloyl glucose	605.1186	453.1073, 313.0541, 169.0140	30 ± 9	27 ±5	-
14	5.130	Quercetin-galactoside-gallate	615.1025	463.0895, 373.0714, 300.0281, 271.0260	148.2 ± 0.3	115 ± 4	-
15	5.222	Isorhamnetin-hexoside	477.0711	315.0149, 299.9903	41 ± 1	27 ± 6	-
16	5.346	Quercetin-glucuronide	477.0659	301.0346	407 ± 15	394 ± 12	-
17	5.941	Methyl-ellagic acid-pentose	447.0954	315.0156, 301.0369	44 ± 5	13 ± 6	-
18	5.992	Quercetin-hexoside	463.0917	301.0361, 258.0544, 179.9977, 151.0038, 107.0140	16 ± 1	19 ± 7	-
19	6.217	Isorhamnetin-rhamnoside	461.0763	315.0158, 301.0319	24 ± 4	15 ± 9	-
20	7.116	Quercetin	301.0376	178.9981, 151.0038, 121.0302, 107.0142	31 ± 2	48 ± 8	-
21	7.459	Dimethyl-hesperetin	329.0331	314.0068, 298.9847, 285.0033	7 ± 1	8 ± 3	0.6 ± 0.1
22	7.695	Naringenin	271.0628	151.0036, 119.0506	3.2 ± 0.7	-	1.4 ± 0.4
23	7.816	Cypellocarpin C	519.1903	335.0768, 233.0436	5.7 ± 1.5	-	-

* Abundance values expressed as ×10^3^; HHDP: hexahydroxydiphenoyl; (-): Not detected.

**Table 4:** Phenolic compounds identified from PLE-NaDES extracts of *S. officinalis* with their respective abundances obtained in the HPLC-QTOF-MS analysis, and permeable and non-permeable compounds through the BBB from the PLE-NaDES extract.

ID	RT (min)	Proposed Compound	*m*/*z * [M-H]^−^	Main Fragment Ions	PLE-NaDES *	Permeable Fraction *	Non-Permeable Fraction *
1	2.031	3,4-dihydroxyphenyl lactic acid “danshensu”	197.0417	179.0388, 135.0447, 123.0464	17 ± 2	12 ± 6	-
2	3.121	Chicoric acid	473.1216	293.0871, 179.0353, 161.0241, 135.0449	59 ± 28	75 ± 12	-
3	3.298	Cafeic acid O-hexoside	341.0820	281.0664, 251.0569, 233.0462, 179.0352, 161.0235	65 ± 12	107 ± 12	-
4	3.406	Quinic acid	191.0526	126.6511	16 ± 2	-	-
5	3.441	Cafeoylquinic acid	353.0815	191.0563, 178.0511, 164.6262	88 ± 2	-	-
6	3.540	Caffeic acid	179.0320	164.9249, 135.0461, 118.0360, 117.0345, 107.0500, 89.0399,	225 ± 10	23 ± 2	7 ± 1
7	3.768	*p*-Coumaric acid	163.0376	119.0388	19.8 ± 0.7	12 ± 2	-
8	3.959	Medioresinol	387.1586	207.1027, 163.1117	271 ± 11	306 ± 26	-
9	4.101	Salvianic acid C	377.0810	179.0352, 161.0245, 135.0455	117 ± 2	197 ± 14	-
10	4.725	Lithospermic acid	537.0933	493.1153, 357.0586, 313.0750, 295.0617, 179.0350, 135.0440	104 ± 4	-	-
11	4.915	Quercetin-glucuronide	477.0584	301.0358	53 ± 3	43 ± 18	-
12	5.248	Rosmarinic acid glucoside	521.1198	429.0422, 359.0976, 311.0569, 161.0247	8.6 ± 0.5	-	-
13	5.683	Isorhamnetin-hexoside	477.0951	462.0713, 323.0740, 315.0700, 300.0216	93 ± 7	-	-
14	5.870	Apigenin-rutinoside	577.1461	269.046	8.3 ± 0.6	-	-
15	5.905	Sagerinic acid	719.1470	359.0771, 197.0458, 179.0342, 161.0241	174 ± 94	171 ± 39	-
16	6.076	Rosmarinic acid	359.0710	197.5398, 179.0344, 161.0240, 151.0385, 133.0292	301 ± 35	312 ± 12	1.07 ± 0.03
17	7.091	Ethyl caffeate	207.0630	179.0345, 161.0238, 135.0449	120 ± 5	79 ± 3	0.75 ± 0.05
18	7.991	Apigenin	269.0410	151.0024, 117.0347	13 ± 4	16 ± 6	12 ± 9
19	8.098	Dimethylrosmarinic acid	387.1387	179.0341, 161.0253, 135.0460	15 ± 5	26 ± 3	4 ±1
20	8.521	Epirosmanol	345.1653	301.1816, 283.1712	198 ± 12	108 ± 16	26 ± 7
21	8.749	Dimethylquercetin	329.1348	314.1499, 301.1833, 287.2003, 179.0319, 161.0253, 151.0769, 133.0289, 119.0347	8 ± 1	-	-
22	9.222	Rosmadial	343.1497	315.1580, 299.1693, 285.1906, 256.1099, 243.1050, 227.1169	12 ± 1	7.7 ± 0.4	10 ± 3
23	9.318	Carnosol	329.1710	285.1876	1465 ± 51	309 ± 68	30 ± 6
24	9.623	Carnosic acid	331.1860	287.2031, 271.1711, 189.0928, 157.0673	1443 ± 247	12 ±1	62 ± 13
25	9.820	Methyl carnosate	345.2018	301.2168, 286.1940, 271.1709, 257.1541	1264 ± 22	20 ± 8	635 ± 56

* Abundance values expressed as ×10^3^; (-): Not detected.

## Data Availability

The original contributions presented in this study are included in the article/Supplementary Materials; further inquiries can be directed to the corresponding authors.

## Supplementary Materials

The mass spectra of the main terpenoids and phenolic compounds in the extracts determined by GC-MS and HPLC-MS, respectively, can be downloaded at: https://www.mdpi.com/article/10.3390/ijms26020601/s1
