# Supplementary material for: Sequential Obtention of Blood–Brain Barrier-Permeable Non-Polar and Polar Compounds from Salvia officinalis L. and Eucalyptus globulus Labill. with Neuroprotective Purposes
Source: Int J Mol Sci. 2025 Jan 12;26(2):601. doi: 10.3390/ijms26020601 (PMC11765258; doi:10.3390/ijms26020601)
Supplement: Supplementary file 1 [file ijms-26-00601-s001.zip › ijms-3417369-supplementary.pdf]

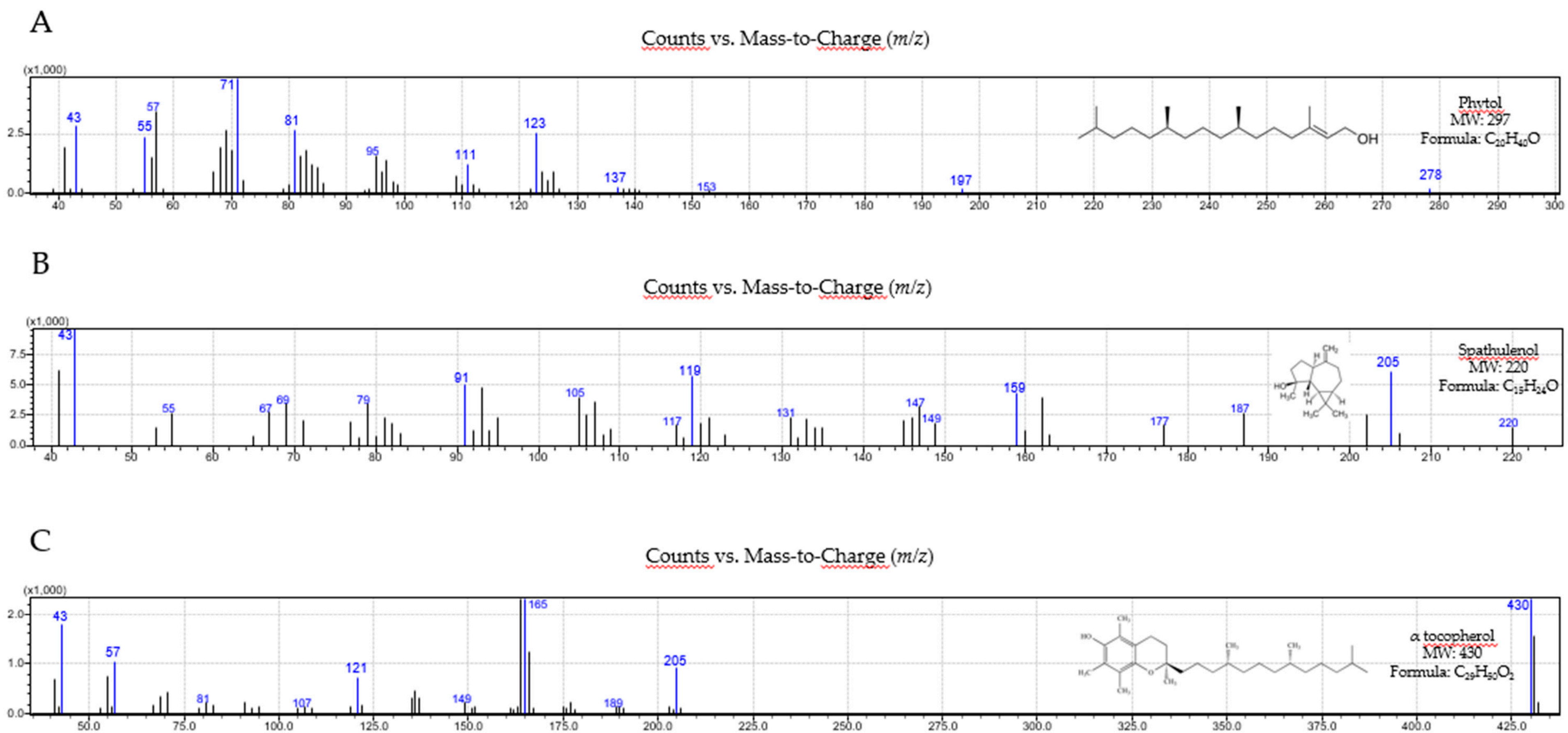

Figure S1. Mass spectra of the major compounds identified in *Eucalyptus globulus* Labill by GC-MS. A) Phytol, B) Spathulenol, C) *α* tocopherol.

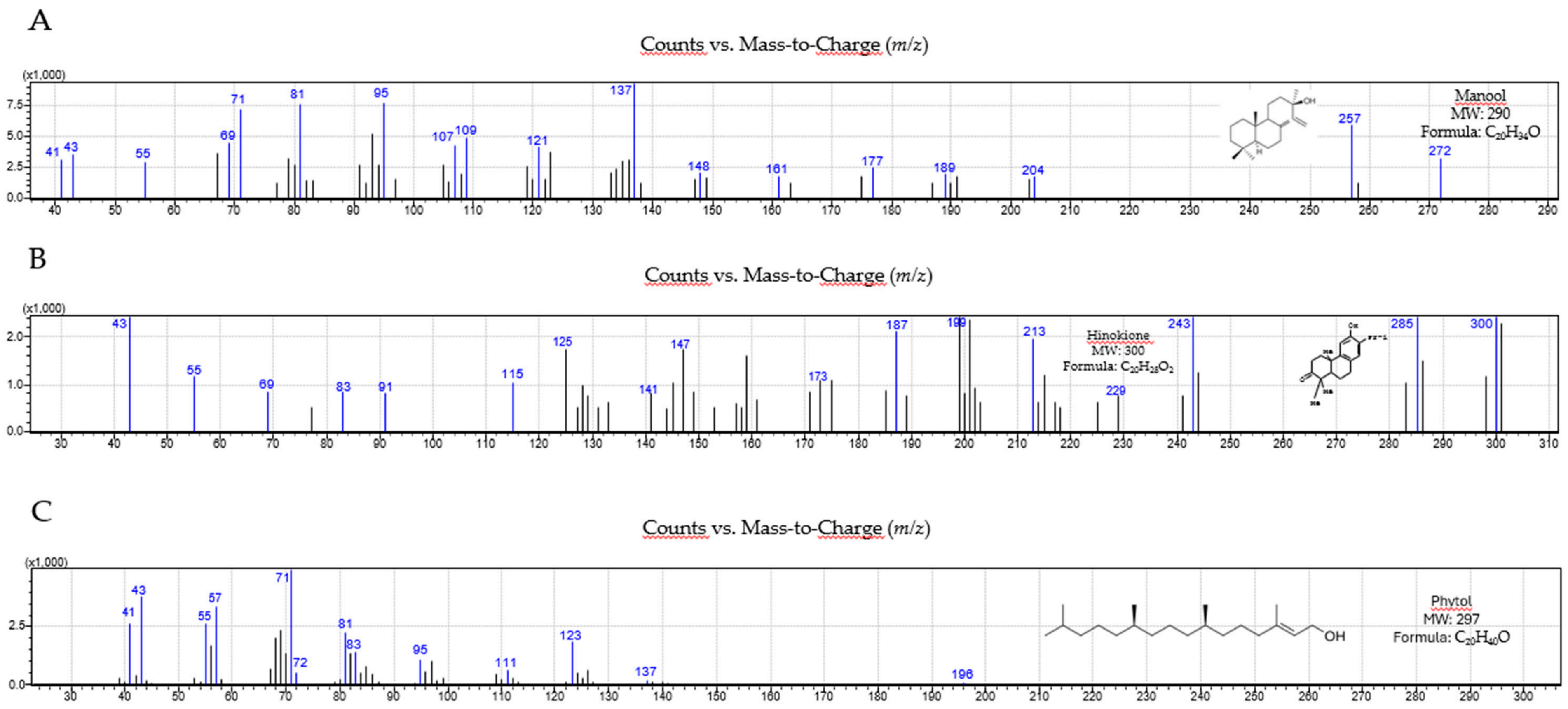

Figure S2. Mass spectra of the major compounds identified in *Salvia officinalis* L by GC-MS. A) Manool, B) Hinokione, C) Phytol.

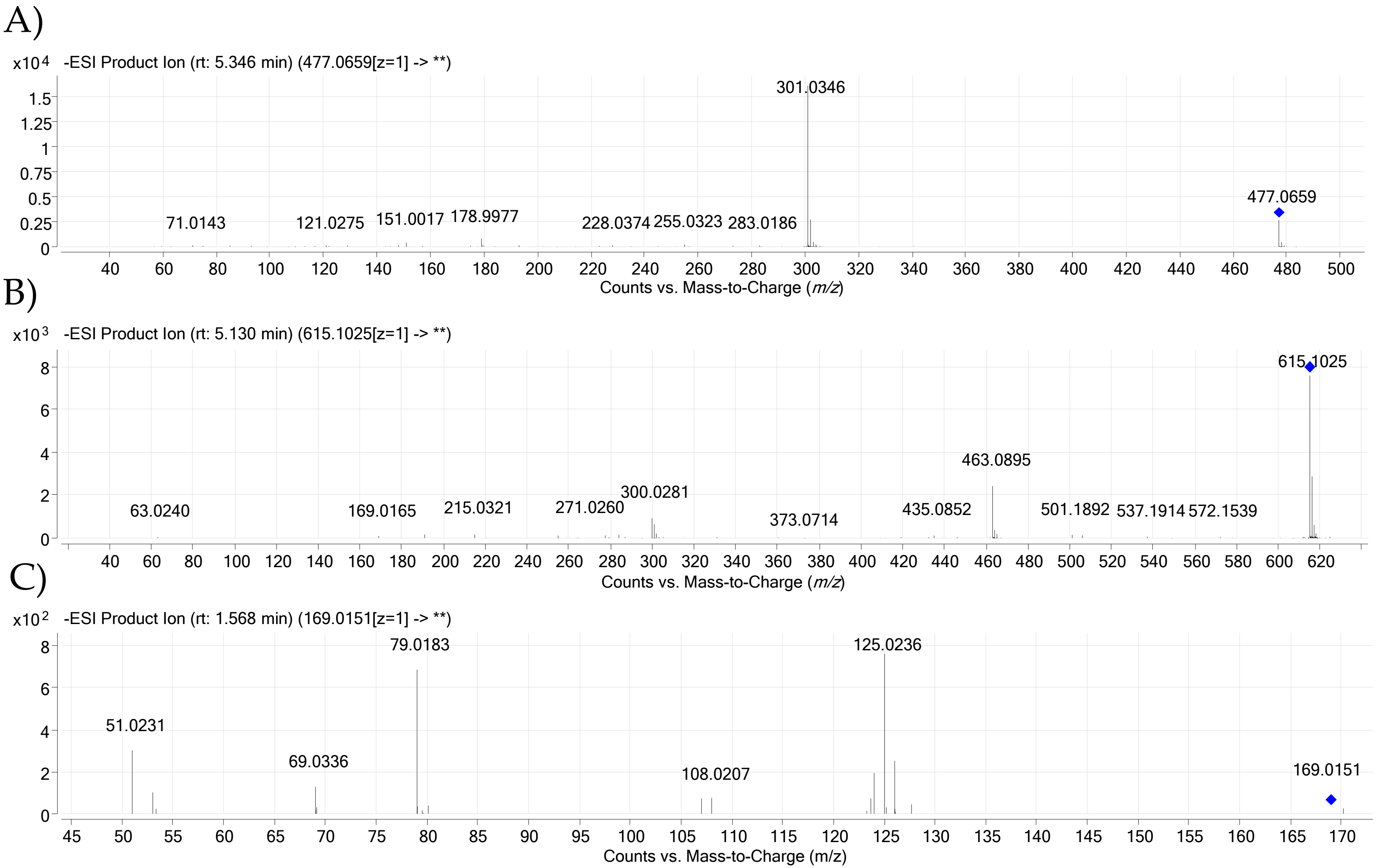

Figure S3. Mass spectra of the major phenolic compounds identified in *Eucalyptus globulus* Labill by HPLC-DAD-QTOF-MS. A) Quercetin-glucuronide, B) Quercetin-galactoside-gallate, C) Gallic acid.

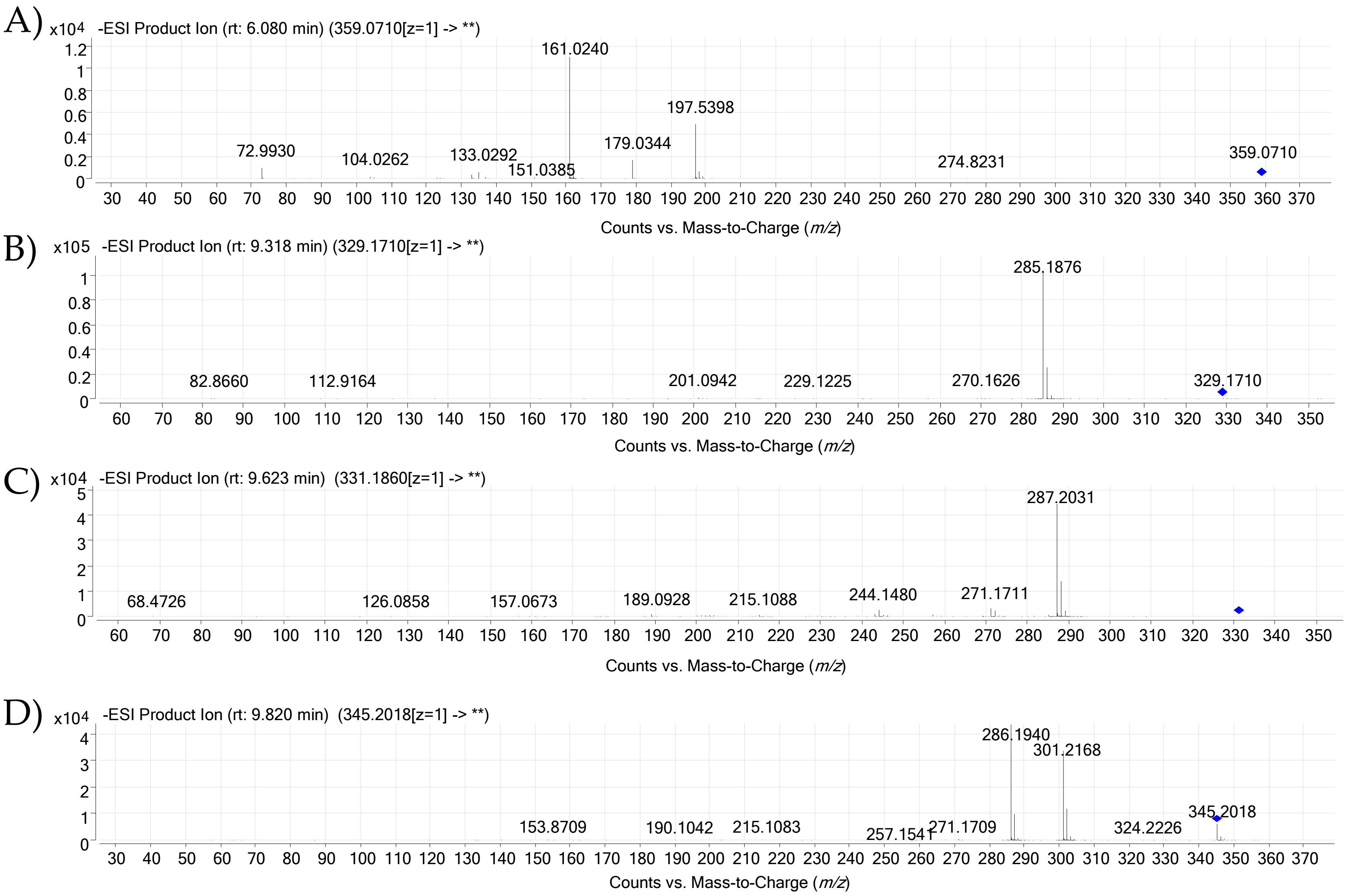

Figure S4. Mass spectra of the major phenolic compounds identified in *Salvia officinalis* L. by HPLC-DAD-QTOF-MS. A) Rosmarinic acid, B) Carnosol, C) Carnosic acid, D) Methyl carnosoate.
